# Sarcopenia‐related changes in serum GLP‐1 level affect myogenic differentiation

**DOI:** 10.1002/jcsm.13524

**Published:** 2024-06-26

**Authors:** Hsien‐Hao Huang, Yun‐Jie Wang, Hui‐Yu Jiang, Helen Wenshin Yu, Yin‐Quan Chen, Arthur Chiou, Jean‐Cheng Kuo

**Affiliations:** ^1^ Department of Emergency Medicine Taipei Veterans General Hospital Taipei Taiwan; ^2^ Institute of Emergency and Critical Care Medicine, School of Medicine National Yang Ming Chiao Tung University Taipei Taiwan; ^3^ Institute of Biochemistry and Molecular Biology National Yang Ming Chiao Tung University Taipei Taiwan; ^4^ Cancer and Immunology Research Center National Yang Ming Chiao Tung University Taipei Taiwan; ^5^ Institute of Biophotonics National Yang Ming Chiao Tung University Taipei Taiwan

**Keywords:** Sarcopenia, GLP‐1, Microtubule motor kinesin‐1, Glucose uptake, ATP production

## Abstract

**Background:**

Sarcopenia, a group of muscle‐related disorders, leads to the gradual decline and weakening of skeletal muscle over time. Recognizing the pivotal role of gastrointestinal conditions in maintaining metabolic homeostasis within skeletal muscle, we hypothesize that the effectiveness of the myogenic programme is influenced by the levels of gastrointestinal hormones in the bloodstream, and this connection is associated with the onset of sarcopenia.

**Methods:**

We first categorized 145 individuals from the Emergency Room of Taipei Veterans General Hospital into sarcopenia and non‐sarcopenia groups, following the criteria established by the Asian Working Group for Sarcopenia. A thorough examination of specific gastrointestinal hormone levels in plasma was conducted to identify the one most closely associated with sarcopenia. Techniques, including immunofluorescence, western blotting, glucose uptake assays, seahorse real‐time cell metabolic analysis, flow cytometry analysis, kinesin‐1 activity assays and qPCR analysis, were applied to investigate its impacts and mechanisms on myogenic differentiation.

**Results:**

Individuals in the sarcopenia group exhibited elevated plasma levels of glucagon‐like peptide 1 (GLP‐1) at 1021.5 ± 313.5 pg/mL, in contrast to non‐sarcopenic individuals with levels at 351.1 ± 39.0 pg/mL (*P* < 0.05). Although it is typical for GLP‐1 levels to rise post‐meal and subsequently drop naturally, detecting higher GLP‐1 levels in starving individuals with sarcopenia raised the possibility of GLP‐1 influencing myogenic differentiation in skeletal muscle. Further investigation using a cell model revealed that GLP‐1 (1, 10 and 100 ng/mL) dose‐dependently suppressed the expression of the myogenic marker, impeding myocyte fusion and the formation of polarized myotubes during differentiation. GLP‐1 significantly inhibited the activity of the microtubule motor kinesin‐1, interfering with the translocation of glucose transporter 4 (GLUT4) to the cell membrane and the dispersion of mitochondria. These impairments subsequently led to a reduction in glucose uptake to 0.81 ± 0.04 fold (*P* < 0.01) and mitochondrial adenosine triphosphate (ATP) production from 25.24 ± 1.57 pmol/min to 18.83 ± 1.11 pmol/min (*P* < 0.05). Continuous exposure to GLP‐1, even under insulin induction, attenuated the elevated glucose uptake.

**Conclusions:**

The elevated GLP‐1 levels observed in individuals with sarcopenia are associated with a reduction in myogenic differentiation. The impact of GLP‐1 on both the membrane translocation of GLUT4 and the dispersion of mitochondria significantly hinders glucose uptake and the production of mitochondrial ATP necessary for the myogenic programme. These findings point us towards strategies to establish the muscle–gut axis, particularly in the context of sarcopenia. Additionally, these results present the potential of identifying relevant diagnostic biomarkers.

## Introduction

Sarcopenia, a category of muscle‐related diseases, is primarily characterized by a decline in muscle mass, strength and function. This geriatric ailment can result in the loss of mobility and an increased risk of bone fracture [S1]. Globally, approximately 10–16% of the elderly population is affected by sarcopenia [S2]. In 2010, the European Working Group on Sarcopenia in Older People (EWGSOP) introduced the first and the most widely accepted set of guidelines, establishing specific thresholds for muscle strength, muscle mass and physical performance to diagnose sarcopenia.^1^ Subsequently, both the International Working Group on Sarcopenia (IWGS)^2^ and the American Foundation for the National Institutes of Health (FNIH) Sarcopenia Project^3^ released their consensus statements to aid the timely diagnosis of sarcopenia in clinical practice. Considering variations in ethnicity, genetic background and body size,^4^ the Asian Working Group for Sarcopenia (AWGS) developed diagnostic criteria tailored to the Asian population.^5^ While these criteria emphasize low muscle strength, mass and quality as the primary clinical indicators of sarcopenia, it is essential to recognize that age‐related changes in muscle tissue architecture and metabolism play a pivotal role in defining the effectiveness of preventive and therapeutic strategies for sarcopenia.

In individuals with sarcopenia, notable pathological alterations in muscle tissue, such as the reduction in muscle mass and the deterioration of satellite cells, which hinder the process of muscle regeneration, could be observed. Muscle fibres (muscle cells), the building blocks of muscle tissue, are formed as myoblasts differentiate into myocytes and then fuse to form myotubes. The primary role of muscle fibres is to generate force and to contract, thereby providing support and enabling the body movement by engaging with their surrounding extracellular matrix. The contractile mechanism within muscle fibres is powered by various cytoskeleton structures, including actin filaments, microtubules and intermediate filaments [S3, S4]. Within muscle cells, microtubules undergo extensive remodelling and align parallel to the primary cell axis. This alignment is crucial in determining the overall shape of a cell during myogenesis. One specific microtubule motor protein, KIF5B, which is the predominant isoform of kinesin‐1 heavy chain in skeletal muscle,^6^ plays a pivotal role in muscle development.^7^ Research via mouse model with a conditional knockout of *Kif5b* in skeletal muscles has demonstrated that the absence of kinesin‐1 leads to the aggregation of nuclei, mitochondria and myofibril components within the cell body, resulting in impaired myofibril assembly and disruptions in muscle fibre terminations.^7^ Furthermore, kinesin‐1 motor proteins are known to facilitate the transportation of mitochondria^8^, ^9^ and glucose transporters (GLUTs).^10^ These processes play a crucial role in regulating energy metabolism and glucose homeostasis. Skeletal muscle, in its normal state, is responsible for approximately 80% of glucose uptake from an oral glucose load^11^ and accounts for 60% of the total body's oxygen consumption required to produce adenosine triphosphate (ATP)^12^ for muscle contractions. Consequently, it becomes imperative to investigate whether systemic metabolic irregularities impact energy metabolism and glucose homeostasis, leading to alterations in muscle tissue metabolism and structure, and ultimately linked to the development of sarcopenia.

Recognizing the significant role of the gastrointestinal environment in maintaining both metabolic balance^13^ and muscle mass,^14^, ^15^ we focused our research on gastrointestinal hormones that could potentially influence muscle metabolism and quality. A prior study applied the criteria established by the EWGSOP^1^ to categorize individuals with sarcopenia, revealed higher levels of various gastrointestinal hormones in this group.^16^ However, as the EWGSOP criteria may not be universally applicable, especially for Asians, we opted to employ the AWGS criteria with adjusted thresholds for the Asian population.^17^ Our investigation revealed a notable increase in the plasma concentration of glucagon‐like peptide 1 (GLP‐1) in individuals affected by sarcopenia. GLP‐1 is a hormone released from L cells in the lower intestine and colon following meals, but it is rapidly degraded by dipeptidyl peptidase‐4 (DPP‐4) in the bloodstream.^18^ It is recognized for its role in stimulating insulin secretion and effectively lowering blood glucose levels. In clinical practice, GLP‐1 receptor (GLP‐1R) agonists are used as medications to treat diabetes by enhancing GLP‐1R signalling and promoting insulin release from pancreatic islets.^19^ While GLP‐1 is acknowledged for its capacity to stimulate pancreatic β‐cell proliferation and insulin secretion, its direct impact on muscle cells and their regulation of myogenic differentiation remains uncertain. In normal individuals, GLP‐1 is reduced within a short period of time, but we observed a significantly higher level of GLP‐1 in starving individuals with sarcopenia, indicating a potential relationship between GLP‐1 and myogenic differentiation in the development of sarcopenia. In this study, we present evidence that exogenous GLP‐1 has a direct detrimental effect on myogenic differentiation. We explored how GLP‐1 inhibits the translocation of GLUT4 on the cell surface, diminishing glucose uptake efficiency, as well as its influence on the distribution of mitochondria, resulting in reduced ATP production during myogenic differentiation. Our research delves into whether higher GLP‐1 levels are linked to impaired myogenic differentiation, and potentially contribute to the development of sarcopenia. Understanding the impact of GLP‐1 in the context of sarcopenia holds promise for identifying the corresponding diagnostic biomarkers.

## Methods

### Cell culture

Mouse myoblasts C2C12, kindly provided by Dr. Pei‐Ching Chang (National Yang Ming Chiao Tung University, Taiwan), were confirmed to be free of mycoplasma contamination and cultured in Dulbecco's Modified Eagle Medium (DMEM)‐high glucose (ThermoFisher) supplemented with 10% fetal bovine serum (ThermoFisher) and 1% penicillin/streptomycin (ThermoFisher). The differentiation of C2C12 cells was conducted following previously described methods.^20^ The induction medium for myogenesis consisted of DMEM‐high glucose supplemented with 2% horse serum and 1% penicillin/streptomycin. In all experiments, the cells were seeded on fibronectin (Jia‐Sheng Cell Tech.) ‐coated coverslips and plates.

### Plasmids and reagents

Antibodies: rabbit anti‐Tom20 (Santa Cruz SC‐11415; dilution for immunofluorescence: 1/50), rabbit anti‐GLUT1 (Invitrogen PA1‐46152; dilution for flow cytometry: 1/1000; dilution for western blotting: 1/1000; dilution for immunofluorescence: 1/500), rabbit anti‐GLUT4 (Abcam Ab33780; dilution for flow cytometry: 1/1000; dilution for immunofluorescence: 1/500; dilution for western blotting: 1/1000), mouse anti‐MYH1/2 (Santa Cruz Sc‐53088; dilution for western blotting: 1/500), rabbit anti‐GAPDH (GeneTex GTX100118; dilution for western blotting: 1/5000), mouse anti‐myosin 4 (the MF20 monoclonal antibody recognizes MYH II; ThermoFisher 14–6503‐82; dilution for immunofluorescence: 1/1000), Alexa Fluor 568 phalloidin (Invitrogen A12380; dilution for immunofluorescence: 1/500), Alexa Fluor 488‐anti‐rabbit IgG (Invitrogen A11034; dilution for immunofluorescence and flow cytometry: 1/300), Alexa Fluor 568‐anti‐mouse IgG (Invitrogen A11031; dilution for immunofluorescence: 1/300), DAPI (Invitrogen D1306; dilution for immunofluorescence: 1/300), Goat anti‐mouse IgG (HRP) (GeneTex GTX213111‐01; dilution for western blotting: 1/10000), Goat anti‐rabbit IgG (HRP) (GeneTex GTX213110‐01; dilution for western blotting: 1/10000).

Reagents: Insulin (Sigma‐Aldrich 12585‐014); QPD‐OTF (Sigma‐Aldrich SML3170); GLP‐1 (Sigma‐Aldrich SI‐G3265).

### Anthropometric indices

Body mass index (BMI) was defined as a measure of weight adjusted for height, calculated as the ratio of body mass in kg and the square of height in meters (kg/m^2^). The sarcopenic index corresponds to the appendicular skeletal muscle mass index (ASMI), which was defined as a measure of appendicular skeletal muscle (ASM) mass adjusted for height, calculated as the ratio of ASM in kg and square of height in meters (kg/m^2^).

### Plasma ghrelin, PYY and GLP‐1 concentrations

Plasma ghrelin, PYY and GLP‐1 concentrations were assessed for all participants. After a fasting period starting at midnight (12:00 am), venous blood sampling from the antecubital vein was carried out between 7:00 and 8:00 am the next morning. Plasma levels of ghrelin, PYY and GLP‐1 were measured using the RayBio Human ELISA kit (Ray Biotech, Norcross, GA, USA). All methods related to humans were carried out under relevant guidelines and regulations. All experiment protocols related to humans were approved by the Ethics Committee of the Institutional Review Board of Taipei Veterans General Hospital. Informed consent was obtained from all subjects.

### Immunofluorescence analysis and image analysis

Mouse C2C12 myoblasts or differentiated C2C12 myotubes were fixed with 4% paraformaldehyde in phosphate‐buffered saline (PBS) at room temperature for 20 min, permeabilized with PBS containing 0.1% saponin at room temperature for 5 min, removed aldehyde groups with 0.1‐M glycine in PBS at room temperature for 10 min and blocked with blocking solution (2% bovine serum albumin [BSA] in PBS) at room temperature for 1 h. Subsequently, the cells were incubated with the indicated primary antibodies in a blocking solution at 4°C for 16 h, and then incubated with fluorescent dye‐conjugated secondary antibodies at room temperature for 1 h. Finally, the cells were mounted on coverslips using a fluorescence mounting medium (DAKO) for epi‐fluorescence imaging. Epi‐fluorescence images were obtained using a microscope (DMRBE, Leica) equipped with a 63×, 1.4NA objective lens (Leica) and a 512B EMCCD (Andor) operated by Micro‐Manager 1.4 software (Leica). Slide‐scanned epi‐fluorescence images were obtained using a microscope (Nikon Eclipse Ti) equipped with a 40×, 1.3NA objective lens (Nikon) and an imaging source (DMK33UX174‐33U) operated by NIS‐Element AR (Nikon).

### Flow cytometry analysis

To assess cell surface expression of glucose transporter 4 (GLUT4) and glucose transporter 1 (GLUT1), cells were washed with PBS and re‐suspended in 2% paraformaldehyde in PBS at room temperature for 15 min. Cells were then blocked with blocking solution (2% BSA in PBS) at room temperature for 1 h, incubated with GLUT4 or GLUT1 antibodies in blocking solution at room temperature for 30 min, washed with PBS, and labelled with Alexa Fluor 488‐anti‐rabbit secondary antibodies at room temperature for 30 min. Cells were then washed, re‐suspended in PBS, and analysed on a CytoFlex FACScan device (Beckman Coulter) operated by CytExpert software (version 2.4, Beckman Coulter). The results were analysed and presented graphically using Excel software (Microsoft).

### Glucose uptake assay

To measure glucose uptake in myotubes, cells were plated on the fibronectin‐coated black 96‐well plates and incubated with myogenic induction medium for 5 days. A glucose uptake cell‐based assay kit (Cayman #600470) was used according to the manufacturer's protocol. In brief, cells were treated with 2% fatty acid‐free BSA in glucose‐free medium (ThermoFisher A1443001) at 37°C for 3 h, and washed with Krebs buffer (126‐mM NaCl, 2.5‐mM KCl, 25‐mM NaHCO_3_, 1.2‐mM NaH_2_PO_4_, 1.2‐mM MgCl_2_, 2.5‐mM CaCl_2_) twice. Fluorescence‐labelled deoxyglucose analog 2‐NBDG was then added to a final concentration of 100 μg/mL in glucose‐free medium at 37°C for 40 min. Free 2‐NBDG was washed away by centrifuging the plate five times at 400 *g* at room temperature. 2‐NBDG taken up by cells was detected with fluorescent filters (excitation/emission = 485/535) using Multimode microplate readers (Tecan Spark). The amount of protein present in each well was used to normalize the data.

### RNA extraction, reverse transcription and real‐time quantitative PCR

RNA was extracted from cells using TRIzol reagent (Invitrogen) and the total RNA precipitated was assessed according to the manufacturer's instructions. The RNA products were reverse‐transcribed using RevertAid First Strand cDNA Synthesis Kits (ThermoFisher) and random hexamer primers. The cDNA products were amplified by PCR using KAPA SYBR® FAST qPCR Kits (ABI Prism; Roche). The target mRNA was quantified by the ΔΔCT method. The qPCR primers for GLUT1 were 5′‐GCTTCTCCAACTGGACCTCAAAC‐3′ and 5′‐ACGAGGAGCACCGTGAAGATGA‐3′. The qPCR primers for GLUT4 were 5′‐CCATCCTGATGACTGTGGCTCT‐3′ and 5′‐GCCACGATGAACCAAGGAATGG‐3′.

### Time‐lapse tracking to assess kinesin‐1 activity and related image analysis

To analyse the transport activity of kinesin‐1 in living C2C12 myotubes, a fluorogenic small molecule substrate QPD‐OTF (Sigma‐Aldrich) was used to track the walking path of kinesin‐1 on microtubules [S6]. Specifically, cells were plated on the fibronectin‐coated coverslips and incubated with a myogenic induction medium for 5 days, washed twice with DMEM (no additives) and put on ice for 1 h. Then the cells were mounted on a magnetic chamber (LCI) and treated with QPD‐OTf (10 μM) in phenol red‐free culture medium with 25 mM Hepes (pH = 7.4). Time‐lapse images of QPD‐OTf were captured at 1‐min intervals using a microscope (Nikon eclipse Ti) equipped with a 40×, 1.3NA objective lens (Nikon) and an imaging source (DMK33UX174‐33U) operated by NIS‐Element AR (Nikon). To analyse the intensity changes of QPD‐OTf, the area of single QPD product precipitates was hand‐outlined over time. The integrated intensities within the areas and backgrounds around the areas were recorded for each time point. The background‐subtracted and photobleach‐corrected intensity values were plotted as a function of time to visualize the motion of kinesin‐1.

### Measurement of ATP production rate

ATP production rate was calculated from oxygen consumption rate (OCR) using a Seahorse XF^e^24 Extracellular Flux Analyzer (Seahorse Bioscience) according to the manufacturer's protocol. C2C12 cells were seeded at a density of 3 × 10^4^ cells per well and cultured in a myogenic induction medium containing dimethyl sulfoxide (DMSO) or GLP‐1 (100 ng/mL) for 5 days before measurement. Oligomycin (1 μM), carbonyl cyanide 4‐(trifluoromethoxy)phenylhydrazone (FCCP; 1 μM) and antimycin A (0.5 μM) with rotenone (0.5 μM) were added sequentially to evaluate mitochondrial respiration. The amount of protein present in each well was used to normalize the data.

### Statistical analysis and data presentation

Statistical significance was calculated by either the Student's *t*‐test or one‐way ANOVA. All the graphs were plotted using Excel software (Microsoft).

## Results

### Endogenous GLP‐1 levels increase in individuals with sarcopenia

**Figure 1:**
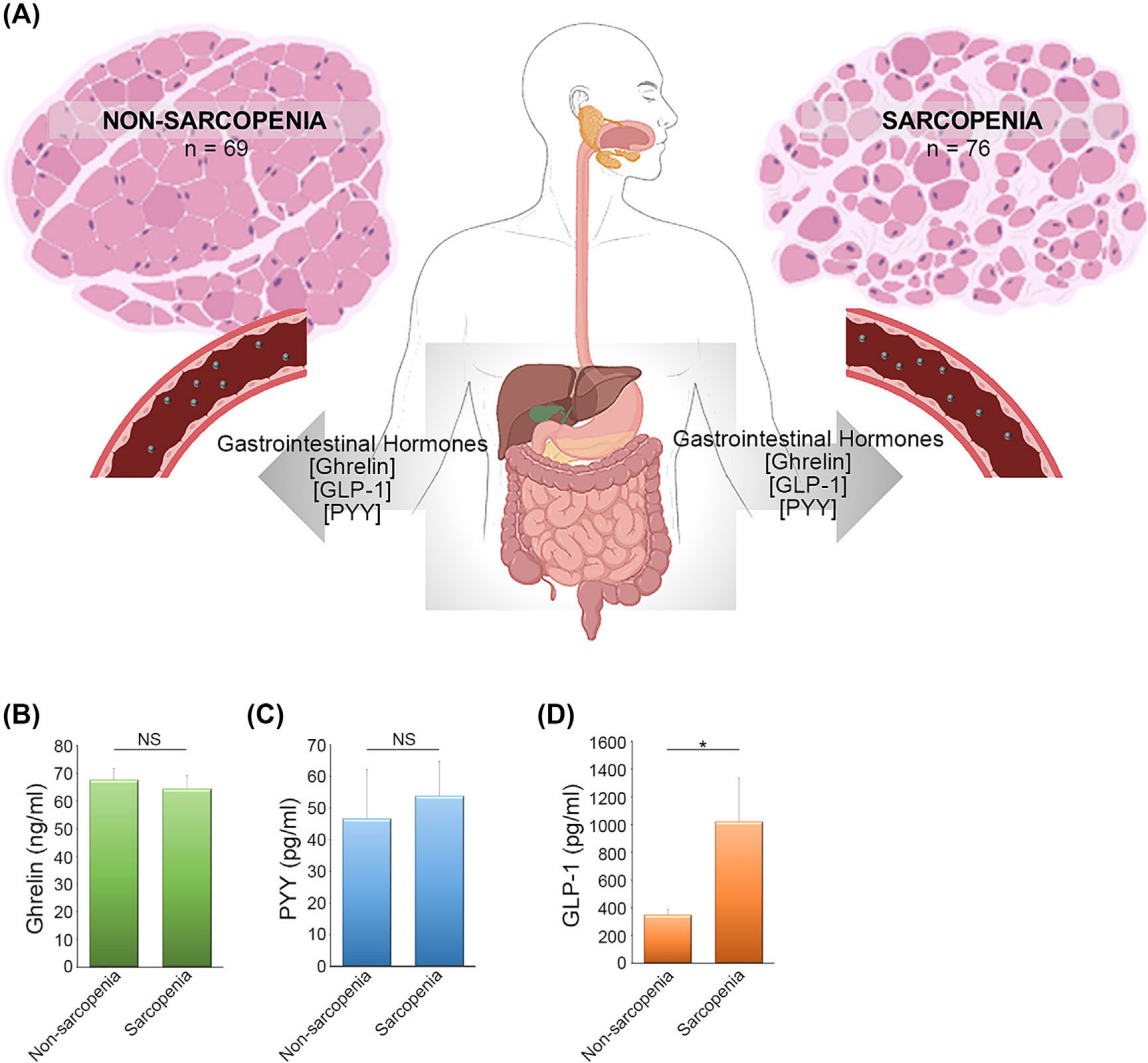
The plasma level of glucagon‐like peptide 1 (GLP‐1) is elevated in individuals with sarcopenia. (*A*) Based on the definition of the Asian Working Group for Sarcopenia (AWGS), 69 individuals were categorized into non‐sarcopenia group and 76 individuals were in sarcopenia group. The plasma levels of gastrointestinal hormones, including ghrelin, GLP‐1 and PYY, were analysed. (*B–D*) The concentrations of (*B*) ghrelin, (*C*) PYY and (*D*) GLP‐1 between individuals in non‐sarcopenia and sarcopenia groups were analysed. Data are mean ± SEM (non‐sarcopenia: *n* = 69 individuals; sarcopenia: *n* = 76 individuals). *P < 0.05.

To ascertain the gastrointestinal hormones that are associated with sarcopenia, a study was conducted involving 145 individuals with an average age of 82.5 ± 0.68 years (*Table* *1*). In accordance with the criteria outlined by the AWGS,^17^ they were categorized into two groups: those with non‐sarcopenia muscle mass and those with sarcopenia. Sarcopenia was diagnosed in individuals who did not meet the handgrip strength requirement during a muscle strength assessment. Of the total participants, 76 were classified as having sarcopenia, while 69 were placed in the non‐sarcopenia group. Notably, the individuals in the sarcopenia group were significantly older than those in the non‐sarcopenia group. Moreover, measurements of height, weight, BMI, arm circumference and waist circumference revealed statistically significant differences, with lower values in the sarcopenia group compared to the non‐sarcopenia group. Bioelectrical impedance analysis (BIA) was applied to examine their body compositions further, focusing on body muscle mass. The results showed that soft lean mass, fat‐free mass and skeletal muscle mass in individuals from the sarcopenia group were significantly lower than those in the non‐sarcopenia group (*Table* *1*). Subsequently, we conducted an analysis of plasma concentrations of the gastrointestinal hormones, including ghrelin, GLP‐1 and PYY, from starving individuals in both groups (*Figure* *1* *A*). No significant differences in the mean blood concentrations of ghrelin (*Figure* *1* *B*) and PYY (*Figure* *1* *C*) were found between the two groups. However, the sarcopenia group exhibited significantly higher plasma GLP‐1 concentrations than the non‐sarcopenia group (*Figure* *1* *D*).

**Table 1:** Comparing patient groups: baseline demographics, clinical assessment variables and comprehensive geriatric assessment

	All	Non‐sarcopenia	Sarcopenia	*P* value
	(*n* = 145)	(*n* = 69)	(*n* = 76)	
Age, years	82.5 ± 0.7	80.3 ± 1.	84.5 ± 0.9	0.002**
Male	88 (60.7%)	44 (63.8%)	44 (57.9%)	
Female	57 (39.3%)	25 (36.2%)	32 (42.1%)	
Height, cm	158 ± 0.7	159.9 ± 1.0	156.2 ± 0.1	0.010*
Weight, kg	59.6 ± 1.1	65.8 ± 1.6	53.9 ± 1.2	<0.001***
Bone mass index, kg/m^2^ (BMI)	23.8 ± 0.4	25.7 ± 0.6	22 ± 0.4	<0.001***
Protein, kg	7.6 ± 0.1	8.5 ± 0.2	6.8 ± 0.2	<0.001***
Fat, kg	19.8 ± 0.8	21.8 ± 1.3	18.1 ± 0.9	0.017*
Soft lean mass, kg	37.3 ± 0.7	41.5 ± 1.0	33.5 ± 0.7	<0.001***
Fat‐free mass, kg	39.7 ± 0.7	44.1 ± 1.0	35.8 ± 0.7	<0.001***
Skeletal muscle mass, kg	20.8 ± 0.4	23.6 ± 0.6	18.4 ± 0.4	<0.001***
Percentage of body fat, %	32.8 ± 0.4	31.9 ± 1.4	32.7 ± 1.2	0.682
Arm circumference, cm	29 ± 0.4	30.8 ± 0.6	27.4 ± 0.4	<0.001***
Waist circumference, cm	84.7 ± 1.2	87.4 ± 1.9	81.7 ± 1.3	0.008**
Visceral fat area, cm^2^	113.4 ± 4.7	115.7 ± 7.6	111.3 ± 5.7	0.641

* *P* < 0.05.

** *P* < 0.01.

*** *P* < 0.001.

### Direct targeting of myoblasts by consistent GLP‐1 treatment for myogenic programme suppression

**Figure 2:**
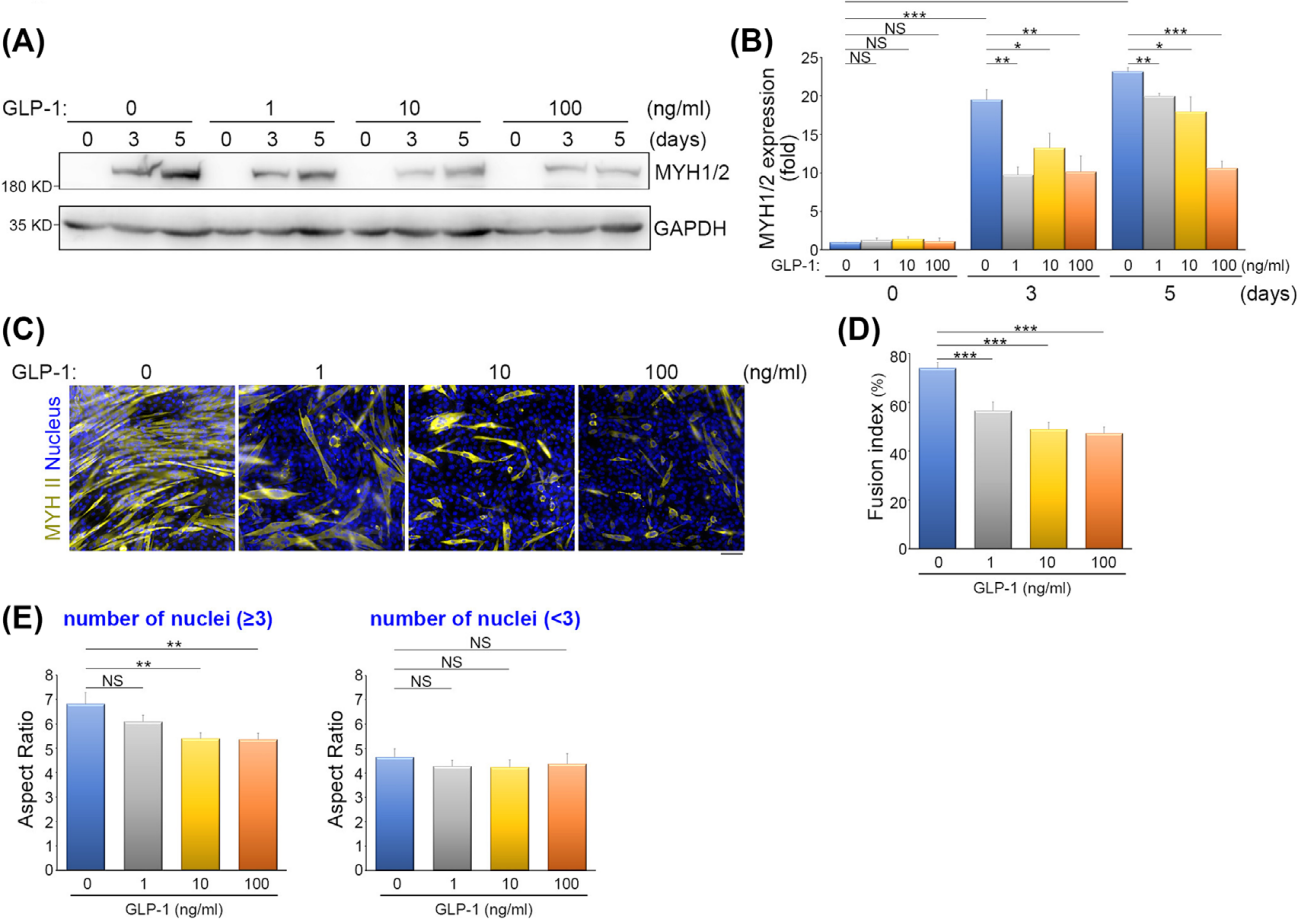
Consistent glucagon‐like peptide 1 (GLP‐1) treatment suppresses the myogenic programme in myoblasts. (*A*) Effects of consistent GLP‐1 treatment on myogenic differentiation. C2C12 cells were treated with a myogenic induction medium containing the indicated concentration of GLP‐1 for 0, 3 and 5 days and analysed by Western blotting using antibodies against MYH1/2 and GAPDH. (*B*) The ratio of MYH1/2 to GAPDH is shown as fold. Data are mean ± SEM (*n* = 3 independent experiments). *P < 0.05; **P < 0.01; ***P < 0.001; NS, no significance. (*C*) Effects of consistent GLP‐1 treatment on myocyte fusion. C2C12 cells were treated with a myogenic induction medium containing the indicated concentrations of GLP‐1 for 5 days and immunostained for myosin 4 (yellow; to visualize MYH II^+^ myotubes) and DAPI (blue; to visualize nucleus). Scale bar, 100 μm. (*D*) Fusion index, calculated as the percentage of nuclei (≥3) in MYH II^+^ cells, as shown in subpart (*C*). Data are mean ± SEM [0 ng/mL GLP‐1: *n* = 15 independent fields (total 620 MYH II^+^ cells counted); 1 ng/mL GLP‐1: *n* = 15 independent fields (total 592 MYH II^+^ cells counted); 10 ng/mL GLP‐1: *n* = 15 independent fields (total 522 MYH II^+^ cells counted); 100 ng/mL GLP‐1: *n* = 12 independent fields (total 511 MYH II^+^ cells counted), from 4 independent experiments]. ***P < 0.001. (*E*) Aspect ratio (the ratio of the longest cell length to the shortest cell length) of MYH II^+^ cells with nuclei <3 or nuclei ≥3, as shown in subpart (*C*). Data are mean ± SEM (*n* = 10 MYH II^+^ cells in each condition). **P < 0.01; NS, no significance.

The loss of skeletal muscle mass is the major definition of sarcopenia, which is caused by a gradual decline in muscle mass, strength and overall functionality [S7–S10]. Muscle regeneration hinges on the activation of muscle stem cells, which possess the capability to differentiate and fuse to facilitate muscle recovery. However, individuals with sarcopenia often exhibit disruptions in the equilibrium of muscle stem cells and impairments in the myogenic programme.^21^ Considering the elevated GLP‐1 levels in starving individuals with sarcopenia (*Figure* *1*), we investigated the potential impact of consistent GLP‐1 treatment on myoblasts and its role in regulating myogenic differentiation. We exposed mouse myoblasts C2C12 cells to myogenic induction media containing varying concentrations of GLP‐1 for 0, 3 and 5 days. We assessed the expression of a myogenic marker MYH1/2 and found that consistent GLP‐1 treatment inhibited the expression of myogenic marker MYH1/2 in the process of myogenic differentiation in a dose‐dependent manner (*Figure* *2* *A,B*). At day 5 of myogenic induction, the cells were stained with MYH II protein and nuclei (*Figure* *2* *C*) to measure myogenic fusion indices (*Figure* *2* *C,D*). Our finding unveiled a dose‐dependent inhibitory effect of consistent GLP‐1 treatment on myocyte fusion (*Figure* *2* *C,D*). Within the population of MYH II^+^ cells, we categorized them into two groups: those with less than three nuclei and those with more than three nuclei, to examine the impact of consistent GLP‐1 treatment on myotubes polarization and elongation. We found that consistent GLP‐1 treatment significantly impeded the aspect ratio of myotubes with more than three nuclei (*Figure* *2* *E*). This observation reveals that consistent GLP‐1 treatment suppressed the elongation of polarized myotubes, which is closely linked to the fusion process, ultimately confirming the capacity of elevated GLP‐1 in reducing the formation of multi‐nuclei myotubes. To ascertain the specificity of consistent GLP‐1 treatment in causing impaired myogenic differentiation, we used PYY as a control, given its stable levels in individuals with sarcopenia under starvation conditions (*Figure* *1*). Our results indicated that consistent PYY treatment had no impact on the myogenic differentiation of myoblasts (*Figure* *S1*).

### Inhibition of glucose uptake during myogenic differentiation by consistent GLP‐1 treatment

**Figure 3:**
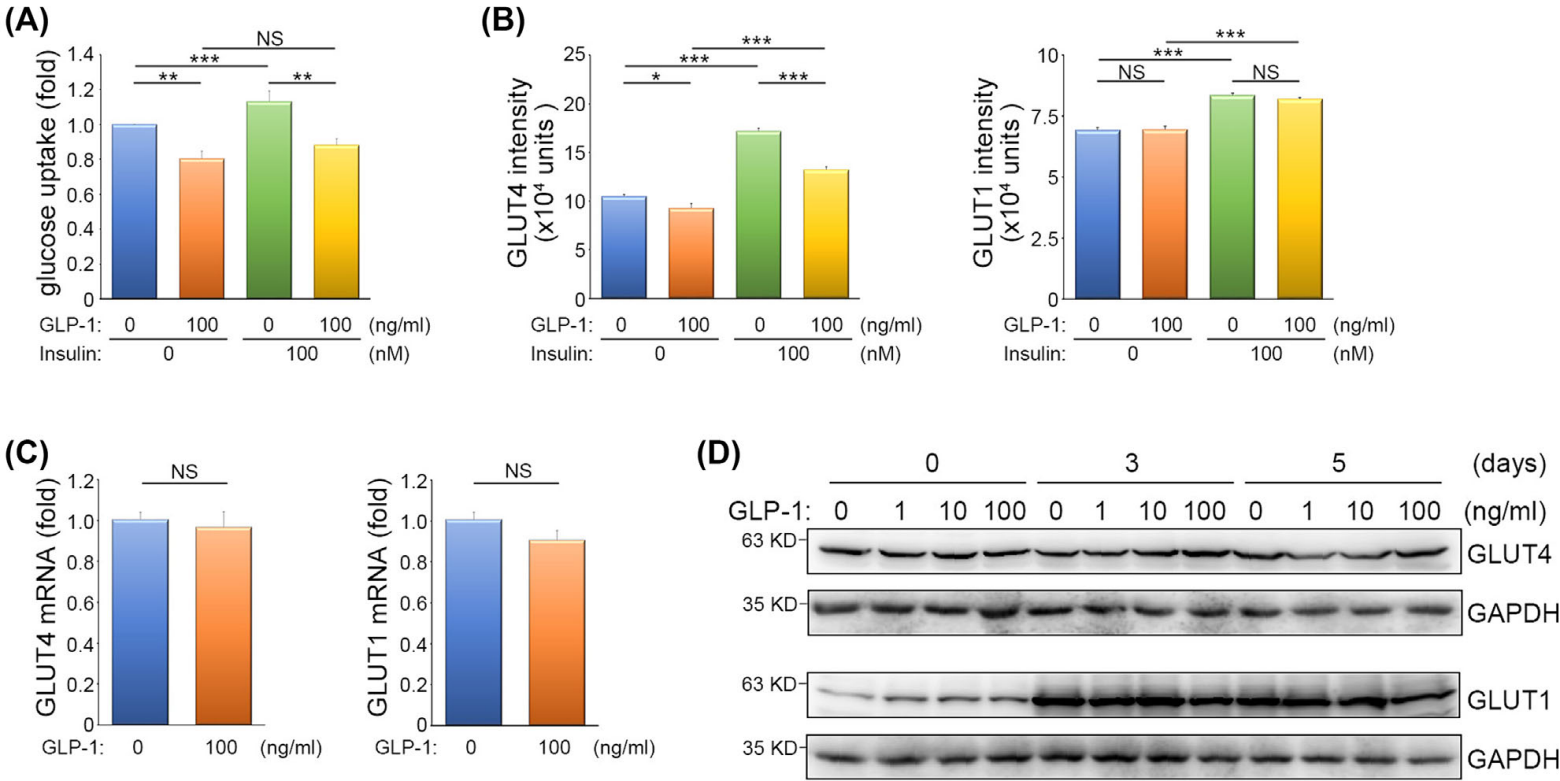
Consistent glucagon‐like peptide 1 (GLP‐1) treatment inhibits glucose transporter 4 (GLUT4) membrane translocation and glucose uptake during myogenic differentiation. (*A*) Effects of consistent GLP‐1 treatment on glucose uptake. C2C12 cells were treated with a myogenic induction medium containing the indicated concentration of GLP‐1 for 5 days. A glucose uptake assay was performed after being treated with or without insulin (100 nM) for 30 min. Data are mean ± SEM [*n* = 4 independent experiments]. **P < 0.01; ***P < 0.001; NS, no significance. (*B*) Effects of consistent GLP‐1 treatment on membrane expression of GLUT4 and glucose transporter 1 (GLUT1). Cells were treated with a myogenic induction medium containing the indicated concentration of GLP‐1 for 5 days and analysed using flow cytometry after being treated with or without insulin (100 nM) for 30 min. Data are mean ± SEM [*n* = 3 independent experiments]. *P < 0.05; ***P < 0.001; NS, no significance. (*C*) Effects of consistent GLP‐1 treatment on GLUT1 and GLUT4 mRNA expression. C2C12 cells were treated with a myogenic induction medium containing GLP‐1 (0 or 100 ng/mL) for 5 days and analysed using real‐time PCR. Data are mean ± SEM (n = 3 independent experiments). NS, no significance. (*D*) Effects of consistent GLP‐1 treatment on GLUT1 and GLUT4 protein expression. C2C12 cells were treated with a myogenic induction medium containing the indicated concentration of GLP‐1 for 0, 3 and 5 days and analysed by Western blotting using antibodies against GLUT1, GLUT4 and GAPDH.

Approximately 80% of the dietary glucose is taken up by skeletal muscle to fuel its performance, primarily through the action of insulin [S11]. While it is known that GLP‐1 can stimulate the pancreas to increase pancreatic β cell mass and insulin secretion,^22^ we sought to investigate whether consistent GLP‐1 treatment could have a direct impact on myotubes, affecting their ability to take up glucose efficiently. After inducing myogenesis and administrating GLP‐1 treatment for 5 days, we observed a substantial impairment in the glucose uptake efficiency of myotubes (*Figure* *3* *A*), revealing a direct effect of GLP‐1 in reducing glucose uptake in myotubes. To delve deeper into how consistent GLP‐1 treatment impeded glucose uptake, we focused on two glucose transporters: glucose transporter 1 (GLUT1) [S12, S13] and glucose transporter 4 (GLUT4) [S14–S18], because their presence on the membrane of muscle cells regulates glucose uptake. Through assessing the fluorescence intensity of GLUT1 and GLUT4 on the surface of differentiated C2C12 myotubes, we discovered that consistent GLP‐1 treatment significantly decreased the expression of surface GLUT4, while leaving GLUT1 unaffected (*Figure* *3* *B*). Even at a concentration of 1 ng/mL, which is relevant in sarcopenia patients, GLP‐1 exhibited an inhibitory effect on GLUT4 membrane translocation (*Figure* *S2*), supporting a dose‐dependent inhibition shown in *Figure* *2* regarding myogenic differentiation. We further investigated their expression at both mRNA level (*Figure* *3* *C*) and protein level (*Figure* *3* *D*) and found that the expression of GLUT1 and GLUT4 remained unchanged following consistent GLP‐1 treatment. These findings indicated that consistent GLP‐1 treatment can directly target myotubes, inhibiting their glucose uptake efficiency by interfering with the translocation of the primary glucose transporter, GLUT4, to the cell membrane.

In the realm of clinical diabetes management, GLP‐1R antagonists and DPP‐4 inhibitors are prescribed medications aimed at stimulating the GLP‐1R signalling pathway. This, in turn, promotes the release of insulin from pancreatic islets.^19^ Subsequently, insulin promotes the translocation of GLUT4 to the cell membrane, facilitating the uptake of glucose.^23^, ^24^ Considering our findings that consistent GLP‐1 treatment contained the capability to affect glucose uptake of myotubes directly (*Figure* *3* *A*), we conducted further investigations to ascertain whether consistent GLP‐1 treatment could hinder insulin's influence on myotube glucose uptake. Following a 30‐min exposure to insulin, we observed a significant enhancement in the efficiency of glucose uptake in myotubes. However, this enhancement was not observed in myotubes consistently treated with GLP‐1 (*Figure* *3* *A*). These outcomes underscored that consistent GLP‐1 treatment inhibited the insulin‐induced glucose uptake within myotubes. Furthermore, we delved into the impact of consistent GLP‐1 treatment on the insulin‐driven translocation of GLUT1 and GLUT4 to the cell membrane. Our observations revealed that insulin significantly promoted the translocation of GLUT1 and GLUT4 to the cell membrane. However, consistent GLP‐1 treatment selectively suppressed the membrane translocation of GLUT4, while leaving GLUT1 unaffected, as induced by insulin (*Figure* *3* *B*). These results emphasized the pivotal role of GLUT4 in myotubes in facilitating glucose uptake, an effect that can be enhanced by insulin but is broadly inhibited by consistent GLP‐1 treatment.

### GLP‐1 inhibits the activity of kinesin‐1 in myotubes

**Figure 4:**
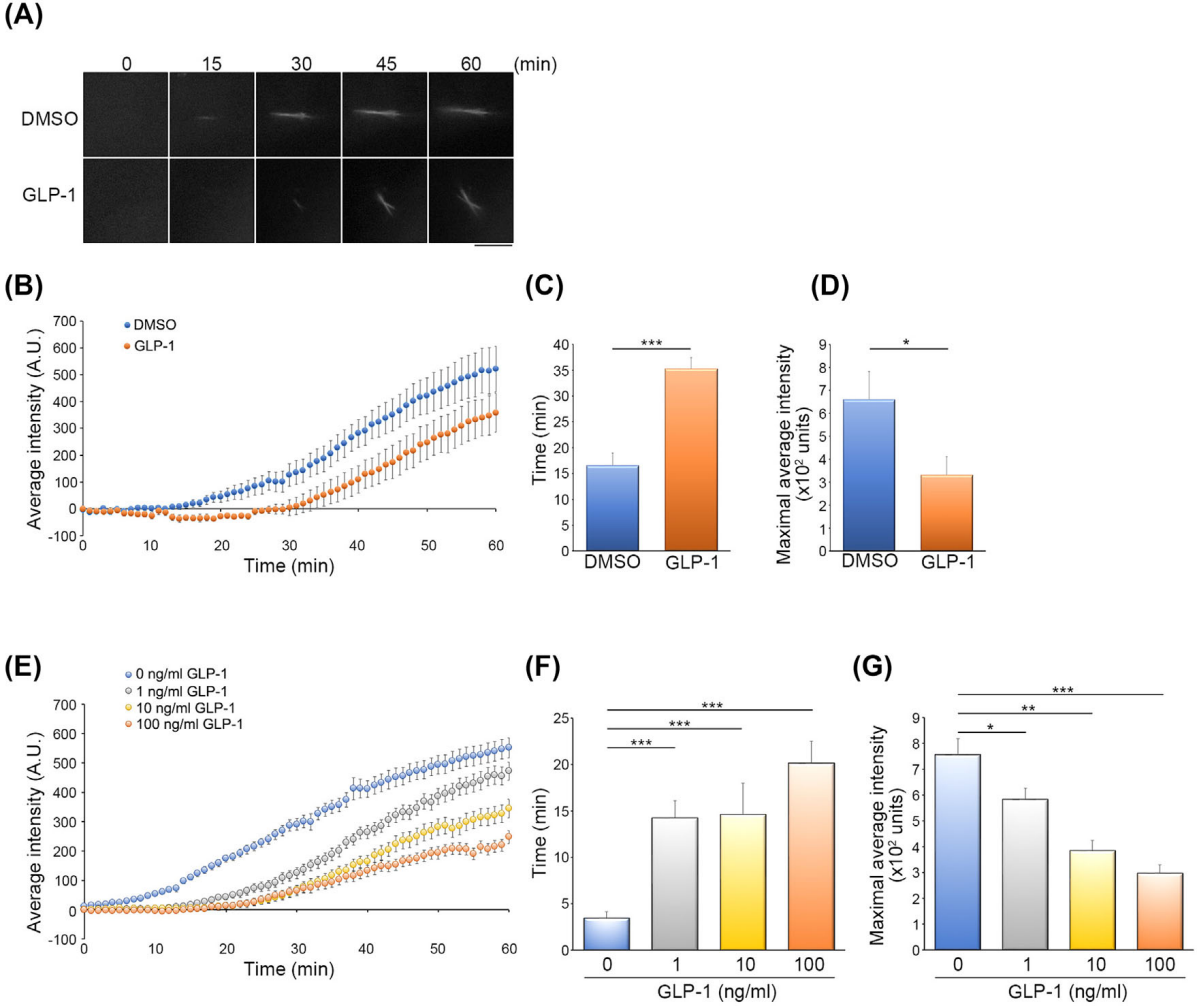
Kinesin‐1 activity is inhibited by consistent glucagon‐like peptide 1 (GLP‐1) treatment in myotubes. (*A–D*) C2C12 cells incubated in a myogenic induction medium containing dimethyl sulfoxide (DMSO) or GLP‐1 (100 ng/mL) for 5 days were treated with QPD‐OTf (10 μM). (*A*) Time‐lapse epi‐fluorescence images showing the effect of consistent GLP‐1 treatment on the formation of QPD crystals in C2C12 myotubes. Scale bar, 10 μm. (B) Average fluorescent QPD crystal intensity (A.U.) over time in C2C12 myotubes. (*C*) The time for the appearance of QPD crystals in C2C12 myotubes. (*D*) The maximal average intensity of QPD crystals in C2C12 myotubes. Data are mean ± SEM. (*n* = 12 QPD crystal in 6 myotubes [DMSO], *n* = 7 QPD crystal in 4 myotubes [GLP‐1]). *P < 0.05; ***P < 0.001. (*E–G*) C2C12 cells incubated in a myogenic induction medium containing GLP‐1 (0, 1, 10 and 100 ng/mL) for 16 h were treated with QPD‐OTf (10 μM). (*E*) Average fluorescent QPD crystal intensity (A.U.) over time in C2C12 myotubes. (*F*) The time for the appearance of QPD crystals in C2C12 myotubes. (*G*) The maximal average intensity of QPD crystals in C2C12 myotubes. Data are mean ± SEM. (*n* = 29 QPD crystal in 7 cells [0 ng/mL], *n* = 27 QPD crystal in 4 cells [1 ng/mL], *n* = 11 QPD crystal in 4 cells [10 ng/mL], *n* = 23 QPD crystal in 5 cells [100 ng/mL]). *P < 0.05; **P < 0.01; ***P < 0.001.

Kinesin‐1, a conventional microtubule motor protein, plays a pivotal role in facilitating the transportation of integrin β1 for the transmission of integrin β1‐mediated FA signals, which are crucial in regulating myogenic differentiation.^20^ There is also evidence pointing to the involvement of kinesin‐1 in enabling the movement of GLUT4 in response to insulin.^10^ Thus, we conducted further investigation to determine whether consistent GLP‐1 treatment impacted the activity of kinesin‐1 in differentiated C2C12 myotubes. We applied live‐cell imaging utilizing soluble pro‐fluorophores called QPD‐OTf, which generates insoluble fluorescent QPD crystal along the path that kinesin‐1 travels on microtubules.^25^ This allowed us to observe and assess the transport activity of kinesin‐1 in living C2C12 myotubes over time. By analysing a time‐lapse image series of QPD crystal formation, we measured the extent of kinesin‐1 activation (*Figure* *4* *A,B*). Our quantitative analysis revealed a significant delay in the onset of QPD crystal accumulation within C2C12 myotubes subjected to continuous GLP‐1 treatment over 5 days, occurring roughly 18 min later than the initiation time observed with DMSO treatment (initiating time of QPD crystal accumulation: 16.55 ± 2.41 min for DMSO‐treated myotubes; 35.33 ± 2.08 min for GLP‐1‐treated myotubes) (*Figure* *4* *C*). Moreover, consistent GLP‐1 treatment resulted in a reduction in the maximum level of QPD crystal accumulation (*Figure* *4* *D*). To delve deeper into whether GLP‐1 exhibited an acute impact on kinesin‐1 activity, C2C12 myoblasts were exposed to GLP‐1 for 16 hours in myogenic induction media. We observed a significant delay in QPD accumulation initiation in a dose‐dependent manner (initiating time of QPD crystal accumulation: 3.45 ± 0.71 min for DMSO treatment; 14.25 ± 1.87 min for 1 ng/mL GLP‐1 treatment; 14.64 ± 3.37 min for 10 ng/mL GLP‐1 treatment; 20.13 ± 2.38 min for 100 ng/mL GLP‐1 treatment) (*Figure* *4* *E,F*). Short‐term GLP‐1 treatment markedly reduced the maximal level of QPD crystal accumulation in a dose‐dependent manner (*Figure* *4* *G*). These results confirmed that GLP‐1 treatment exerts a negative effect on the inherent transport activity of kinesin‐1 in differentiated myotubes.

### Induction of mitochondrial dysfunction in myotubes through continuous GLP‐1 treatment

**Figure 5:**
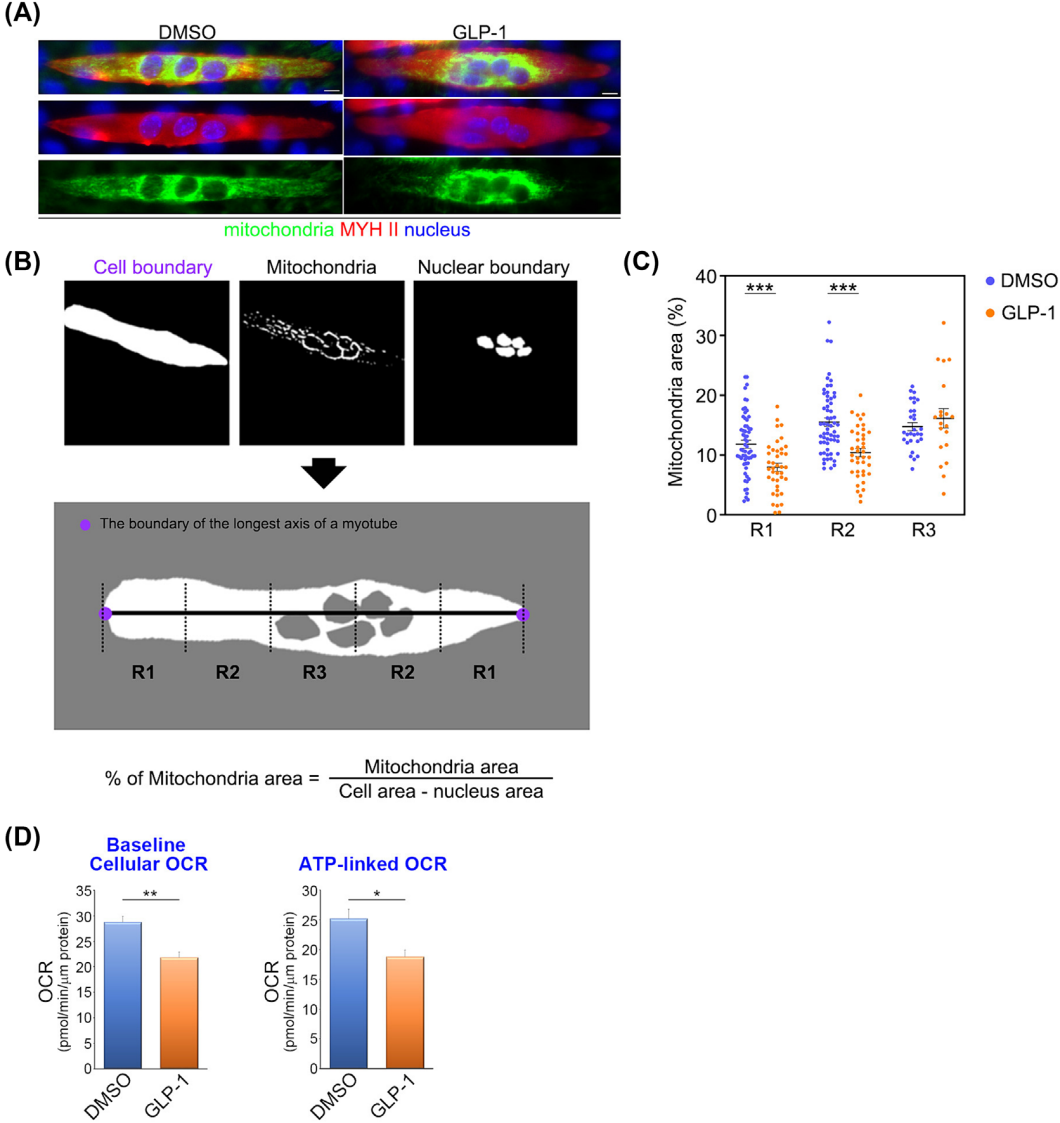
Consistent glucagon‐like peptide 1 (GLP‐1) treatment causes mitochondrial dysfunction in the myogenic programme. (*A*) Images of C2C12 cells treated with a myogenic induction medium containing dimethyl sulfoxide (DMSO) or GLP‐1 (100 ng/mL) for 5 days; these were stained for myosin 4 (red; to visualize MYH II^+^ myotubes), Tom20 (to visualize mitochondria; red) and DAPI (to visualize nucleus; blue). Scale bar, 10 μm. (*B*) Workflow illustrating the steps of categorizing the percentage of mitochondria spreading area (marked with Tom20) into R1, R2, or R3. Using the MYH II^+^ image, the orientation of a myotube was adjusted to align the longest axis of the cell horizontally. Subsequently, the image was divided into five segments based on the average length of the myotube's longest axis. R1 denotes the outermost part, R2 represents the intermediate area and R3 corresponds to the central portion. (*C*) Comparison of the percentage of the mitochondria spreading area as a function within the delineated region in subpart (*B*). Data are mean ± SEM (DMSO: *n* = 30 cells; GLP‐1: *n* = 20 cells, from 3 independent experiments). ***P < 0.001. (*D*) Baseline cellular OCR (oxygen consumption rate) and ATP‐linked OCR were analysed in C2C12 cells treated with a myogenic induction medium containing DMSO or GLP‐1 (100 ng/mL) for 5 days. OCR was measured continuously followed by the addition of oligomycin (1 μM), carbonyl cyanide 4‐(trifluoromethoxy) phenylhydrazone (FCCP; 1 μM) and antimycin A (0.5 μM) with rotenone (0.5 μM) using seahorse XF^e^24 Extracellular Flux Analyzer. Data are mean ± SEM (*n* = 3 independent experiments). *P < 0.05; **P < 0.01.

The activity of kinesin‐1 motor protein is recognized to influence mitochondrial movement.^9^ Therefore, we further investigated the impact of GLP‐1‐induced inhibition of kinesin‐1 activity on mitochondrial distribution during myogenic programming. Utilizing Tom20 staining as a mitochondrial marker, we evaluated the distribution of mitochondria in differentiated C2C12 myotubes subjected to continuous 5‐day treatment with either DMSO or GLP‐1. Our observation revealed a distinct redistribution of the mitochondria towards the geometric centre of GLP‐1‐treated myotubes (*Figure* *5* *A*). To gain further insight into the impact of consistent GLP‐1 treatment on mitochondrial distribution, we categorized mitochondria into three regions (R1, R2 and R3) based on their intracellular localization relative to the longest axis within the myotube (*Figure* *5* *B*). Comparison of mitochondrial redistribution between DMSO‐treated and GLP‐1‐treated C2C12 myotubes showed a significant decrease in the percentage of mitochondrial area within the R1 (outermost) and R2 (intermediate) regions in myotubes consistently exposed to GLP‐1 (*Figure* *5* *C*). This indicates that consistent GLP‐1 treatment has an influence on restricting mitochondrial movement towards the cell periphery. Given the mitochondrial redistribution of mitochondria can affect cellular ATP production,^26^ we further assessed mitochondrial function using Seahorse XF24 Extracellular Flux analysis. Following myogenic differentiation and 5‐day treatment with either DMSO or GLP‐1, we observed a significant reduction in both baseline cellular oxygen consumption rate (OCR) and ATP‐linked OCR in myotubes consistently treated with GLP‐1. These measurements indicate suppressed basal respiration and cellular ATP production (*Figure* *5* *D*). Hence, it is evident that maintaining a continuous supply of GLP‐1 throughout the myogenic differentiation process leads to a constriction of mitochondrial distribution. This shift in mitochondrial distribution induced by consistent GLP‐1 treatment results in mitochondrial dysfunction, including reduced basal respiration and diminished cellular ATP production.

## Discussion

**Figure 6:**
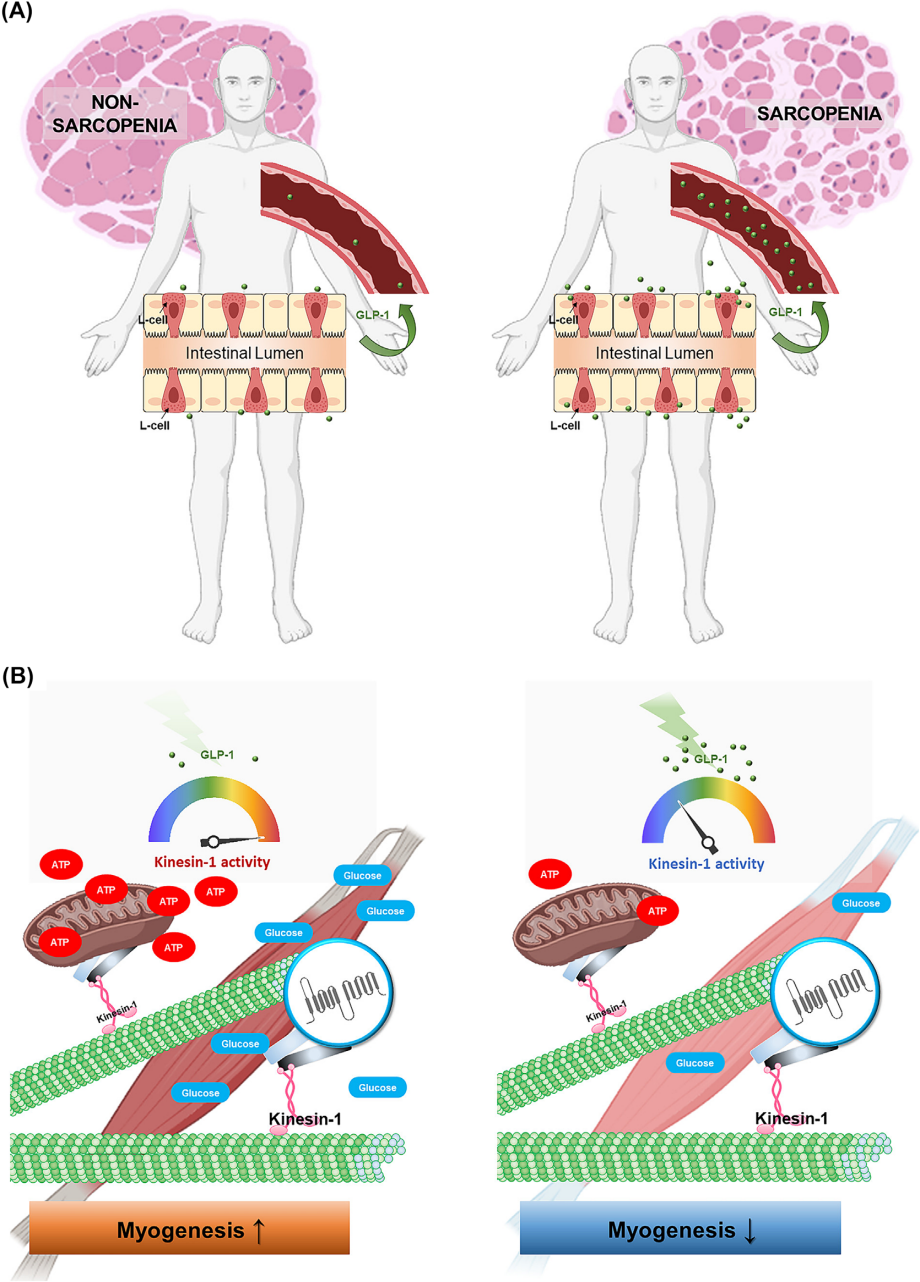
Model of elevated bloodstream glucagon‐like peptide 1 (GLP‐1) in sarcopenia. (*A*) Elevated GLP‐1 levels were detected in the bloodstream of individuals with sarcopenia, in contrast to those without the condition (non‐sarcopenia). (*B*) Continual administration of high‐dose GLP‐1 results in the suppression of kinesin‐1 activity, which inhibits the translocation of glucose transporter 4 (GLUT4) to the cell membrane and the dispersion of mitochondria. This, in turn, hinders glucose uptake and mitochondrial adenosine triphosphate (ATP) production, ultimately contributing to the impairment of myogenic differentiation.

The primary objective of this study was to pinpoint the gastrointestinal hormones linked to sarcopenia and assess their potential to impact myogenic differentiation, with the prospect of identifying them as diagnostic biomarkers. To achieve this goal, we specifically focused on the gastrointestinal hormones, which include ghrelin, PYY and GLP‐1. Our study involved 145 participants, with an average age of 82.5 ± 0.68 years, who were categorized into either a sarcopenia group (consisting of 76 individuals) or a non‐sarcopenia group (consisting of 69 individuals) based on the criteria outlined by the AWGS.^17^ Our finding revealed a notable increase in the levels of GLP‐1 among sarcopenia individuals (*Figure* *6* *A*). This finding aligns with previous research,^16^ which adhered to the EWGSOP definition. Although naturally produced endogenous GLP‐1 is rapidly degraded, our clinical observations have prompted us to hypothesize that elevated GLP‐1 levels may directly interfere with myoblasts, impeding the process of myogenic differentiation and potentially contributing to the onset of sarcopenia. Indeed, we confirmed that consistent GLP‐1 treatment directly impeded the efficiency of myogenic differentiation in C2C12 myoblasts. During myogenic differentiation, we observed that consistent GLP‐1 treatment suppressed the activity of kinesin‐1, a motor protein responsible for transporting GLUT4 to the plasma membrane and facilitating the dispersion of mitochondria. The inhibition of GLUT4 membrane translocation and mitochondria dispersion resulted in impaired glucose uptake and reduced ATP production, ultimately impeding myogenic differentiation (*Figure* *6* *B*). These findings indicate, for the first time, that consistent GLP‐1 treatment can directly target myoblasts, inhibiting kinesin‐1 motor activity and, in turn, impeding the transport of GLUT4 and mitochondria. This disruption leads to decreased efficiency in glucose uptake and ATP production, ultimately contributing to impaired myogenic differentiation. Elucidating the impact of elevated GLP‐1 in sarcopenia patients holds promise as a crucial diagnostic biomarker.

Glucose oxidation plays a significant role in generating energy within skeletal muscle, and its control is influenced by insulin. Insulin is known to enhance glucose uptake by promoting the movement of GLUT4 to the cell membrane,^23^, ^24^ and it stimulates the utilization of glucose in the mitochondria, leading to increased ATP production in muscle cells.^27^ GLP‐1 levels naturally rise after a meal, promoting the pancreas to release insulin, and thus, GLP‐1 acts as an incretin hormone. Naturally, GLP‐1 has a short lifespan in the bloodstream, as it is rapidly degraded by DPP‐4.^18^ However, the elevated levels of GLP‐1 in starving individuals with sarcopenia lead us to believe that GLP‐1 may play a role in the development of this condition, although the precise reason for this elevation remains unclear. Despite the ongoing mystery regarding the higher levels of GLP‐1 in individuals with sarcopenia, we have confirmed that consistent GLP‐1 treatment directly affects myoblasts, hindering the process of myogenic differentiation. Additionally, we have identified that, in the process of myogenic differentiation, consistent GLP‐1 treatment inhibits the activity of kinesin‐1 motor proteins, and further suppresses the movement of GLUT4 to the cell surface and mitochondrial dispersion, resulting in the reduction of glucose uptake and mitochondrial ATP production in muscle cells. Although the exact mechanism by which GLP‐1 interferes with insulin signalling, which is initiated by GLP‐1,^19^ remains unclear, we have ascertained that consistent GLP‐1 treatment overrides insulin signalling, leading to the suppression of insulin‐induced GLUT4 membrane translocation. This suppression, in turn, inhibits insulin‐induced glucose uptake in muscle cells. These findings not only corroborate previous research indicating the association of inactive kinesin‐1 with sarcopenia^28^ but also support our clinical observations, which identify increased GLP‐1 levels in starving individuals with sarcopenia. Although it is uncertain whether the effect of GLP‐1 on kinesin‐1 activity suppression is specific to muscles or a general phenomenon, given the crucial role of kinesin‐1‐mediated GLUT4 membrane translocation in adipocytes,^10^ further studies on GLP‐1's impact on other cell types are warranted in future research.

GLP‐1 exerts its insulinotropic actions by engaging with the GLP‐1R, a type B class G‐protein‐coupled receptor located on pancreatic islet β cells. The presence of GLP‐1R is not limited to the pancreas but has been identified in various other tissues, including the central nervous system, peripheral nervous system, gastrointestinal tract, kidneys, lungs, liver, heart and muscle.^29^ When GLP‐1 binds to GLP‐1R, it initiates a signalling cascade that subsequently leads to the generation of cyclic adenosine monophosphate (cAMP). This, in turn, activates cellular cAMP effectors, such as protein kinase A (PKA), and exchange protein directly activated by cAMP (EPAC).^30^, ^31^ The primary effect of GLP‐1 is stimulating pancreatic β cells; activated PKA directly phosphorylates a regulatory subunit of K^+^ ATP channels,^32^ resulting in channel closure and an increased accumulation of K^+^ ions.^33^ Consequently, this facilitates the influx of Ca^2+^ and promotes insulin secretion.^31^ While the influence of GLP‐1 on skeletal muscle through insulin actions is well‐documented, most studies that explored the direct impact of GLP‐1 on skeletal muscle used GLP‐1R agonists, mainly because GLP‐1 naturally has a very short lifespan in the bloodstream, suggesting limited direct effects on skeletal muscle via the GLP‐1R. However, we have detected elevated levels of GLP‐1 in starving individuals with sarcopenia and an increased level of GLP‐1R mRNA in the tibialis anterior muscle of sarcopenic mice (data not shown). These findings imply a direct influence of GLP‐1 on the skeletal muscle of individuals with sarcopenia through GLP‐1R signalling. While the interaction between GLP‐1 and GLP‐1R primarily results in cAMP production and activating the cAMP‐PKA signalling pathway, cAMP‐dependent signalling pathways have been determined to suppress myogenesis.^34^, ^35^ These findings align with our research, supporting the notion that elevated GLP‐1 in individuals with sarcopenia plays a crucial role in impacting the machinery of myogenesis, and potentially contributes to the development of sarcopenia. Given that silencing GLP‐1R in myoblasts resulted in delayed cell proliferation (data not shown), additional investigation into GLP‐1‐mediated signalling pathways is necessary to comprehend their involvement in impaired myogenic differentiation.

A variety of GLP‐1R agonists have been developed to treat type 2 diabetes mellitus. They are designed to mimic the actions of naturally producing GLP‐1, which engages with GLP‐1R. However, unlike natural GLP‐1, these agonists are not quickly degraded by DPP4,^18^ resulting in sustained therapeutic effects that activate GLP‐1R signalling in pancreatic islet β cells, ultimately leading to insulin secretion. Peptide agonists like exendin‐4 and liraglutide^36^ have been developed and are currently used in clinical practice to treat type 2 diabetes mellitus due to their similarity to the amino acid sequence of GLP‐1. Furthermore, it has been observed that applying these GLP‐1R agonists to skeletal muscle enhances glucose transportation.^37^, ^38^, ^39^ However, their impact on kinesin‐1 activity and the process of myogenesis remains unknown. These GLP‐1R peptide agonists are susceptible to protease degradation, making them ineffective for oral use. In contrast, small molecule GLP‐1R non‐peptide agonists, such as Boc5 and WB4‐24,^40^ are still under investigation to confirm their effectiveness in treating type 2 diabetes mellitus. Interestingly, our discovery of elevated GLP‐1 levels in starving individuals with sarcopenia (*Figure* *1*) implies that these GLP‐1R agonists may potentially contribute to the development of sarcopenia. However, further research is necessary to determine the precise mechanisms behind the increased endogenous production of GLP‐1 in individuals with sarcopenia and to assess whether GLP‐1R agonists influence kinesin‐1 activity in skeletal muscle cells.

## Conflict of interest statement

The authors declare that they have no conflict of interest.

## Supporting information


**Data S1.** Supplemental Figure Captions and References.

**Figure S1.** **Consistent PYY treatment does not affect the myogenic program in myoblasts.** (A) Effects of consistent PYY treatment on myogenic differentiation. C2C12 cells were treated with a myogenic induction medium containing the indicated concentration of PYY for 0, 3 and 5 days and analysed by Western blotting using antibodies against MYH1/2 and GAPDH. (B) The ratio of MYH1/2 to GAPDH is shown as fold. Data are mean ± s.e.m (*n* = 3 independent experiments). ***P < 0.001; NS, no significance. (C) Effects of consistent PYY treatment on myocyte fusion. C2C12 cells were treated with a myogenic induction medium containing the indicated concentrations of PYY for 5 days and immunostained for myosin 4 (yellow; to visualize 10 MYH II + myotubes) and DAPI (blue; to visualize nucleus). Scale bar, 100 μm. (D) Fusion index, calculated as the percentage of nuclei (≥ 3) in MYH II + cells, as shown in (C). Data are mean ± s.e.m (0 ng/ml PYY: *n* = 13 independent fields [total 537 MYH II + cells counted]; 0.1 ng/ml 13 *n* = 10 independent fields [total 368 MYH II + cells counted]; 1 ng/ml PYY: *n* = 6 independent fields [total 339 MYH II + cells counted], from 3 independent experiments). NS, no significance.

**Figure S2. Consistent GLP‐1 treatment inhibits GLUT4 membrane translocation during myogenic differentiation.** (A and B) Effects of consistent GLP‐1 treatment on membrane expression of GLUT4. Cells were treated with a myogenic induction medium containing the indicated concentration of GLP‐1 for 5 days. Subsequently, flow cytometry analysis was conducted using antibodies against GLUT4 or buffer alone (Control), followed by labelling with the Alexa Fluor 488‐conjugated secondary antibody. (A) The representative flow cytometry plots. (B) The average GLUT4 intensity. Data are mean ± s.e.m (*n* = 4 independent experiments). **P < 0.01.
