# Supplementary material for: Sarcopenia‐related changes in serum GLP‐1 level affect myogenic differentiation
Source: J Cachexia Sarcopenia Muscle. 2024 Jun 26;15(5):1708–21. doi: 10.1002/jcsm.13524 (PMC11446708; doi:10.1002/jcsm.13524)
Supplement: Supplementary file 1 — Data S1. Supplemental Figure Captions and References. [file JCSM-15-1708-s003.pdf]

SUPPLEMENTAL FIGURES

Figure S1. Consistent PYY treatment does not affect the myogenic program in myoblasts.

(A) Effects of consistent PYY treatment on myogenic differentiation. C2C12 cells were treated with a myogenic induction medium containing the indicated concentration of PYY for 0, 3, and 5 days and analyzed by Western blotting using antibodies against MYH1/2 and GAPDH. (B) The ratio of MYH1/2 to GAPDH is shown as fold. Data are mean ± s.e.m (n = 3 independent experiments). ***$p < 0.001$; NS, no significance. (C) Effects of consistent PYY treatment on myocyte fusion. C2C12 cells were treated with a myogenic induction medium containing the indicated concentrations of PYY for 5 days and immunostained for myosin 4 (yellow; to visualize MYH II^{+} myotubes) and DAPI (blue; to visualize nucleus). Scale bar, 100 µm. (D) Fusion index, calculated as the percentage of nuclei (≥ 3) in MYH II^{+} cells, as shown in (C). Data are mean ± s.e.m (0 ng/ml PYY: n = 13 independent fields [total 537 MYH II+ cells counted]; 0.1 ng/ml PYY: n = 10 independent fields [total 368 MYH II+ cells counted]; 1 ng/ml PYY: n = 6 independent fields [total 339 MYH II+ cells counted], from 3 independent experiments). NS, no significance.

Figure S2. Consistent GLP-1 treatment inhibits GLUT4 membrane translocation during myogenic differentiation.

(A and B) Effects of consistent GLP-1 treatment on membrane expression of GLUT4. Cells were treated with a myogenic induction medium containing the indicated concentration of GLP-1 for 5 days. Subsequently, flow cytometry analysis was conducted using antibodies against GLUT4 or buffer alone (Control), followed by labeling with the Alexa Fluor 488-conjugated secondary antibody. (A) The representative flow cytometry plots. (B) The average GLUT4 intensity. Data are mean ± s.e.m (n = 4 independent experiments). **$p < 0.01$.

SUPPLEMENTAL REFERENCES

1. Szulc P, Beck TJ, Marchand F, Delmas PD. Low skeletal muscle mass is associated with poor structural parameters of bone and impaired balance in elderly men--the MINOS study. J Bone Miner Res. 2005;20:721-9.
2. Yuan S, Larsson SC. Epidemiology of sarcopenia: Prevalence, risk factors, and consequences. Metabolism. 2023;144:155533.
3. Crawford GL, Horowits R. Scaffolds and chaperones in myofibril assembly: putting the striations in striated muscle. Biophys Rev. 2011;3:25-32.
4. Sanger JW, Wang J, Fan Y, White J, Sanger JM. Assembly and dynamics of myofibrils. J Biomed Biotechnol. 2010;2010:858606.
5. Angerani S, Lindberg E, Klena N, Bleck CKE, Aumeier C, Winssinger N. Kinesin-1 activity recorded in living cells with a precipitating dye. Nat Commun. 2021;12:1463.
6. Cruz-Jentoft AJ, Bahat G, Bauer J, Boirie Y, Bruyere O, Cederholm T, et al. Sarcopenia: revised European consensus on definition and diagnosis. Age Ageing. 2019;48:16-31.
7. Jones TE, Stephenson KW, King JG, Knight KR, Marshall TL, Scott WB. Sarcopenia--mechanisms and treatments. J Geriatr Phys Ther. 2009;32:83-9.
8. Kim TN, Park MS, Lee EJ, Chung HS, Yoo HJ, Kang HJ, et al. Comparisons of three different methods for defining sarcopenia: An aspect of cardiometabolic risk. Sci Rep. 2017;7:6491.
9. Meza-Valderrama D, Marco E, Davalos-Yerovi V, Muns MD, Tejero-Sanchez M, Duarte E, et al. Sarcopenia, Malnutrition, and Cachexia: Adapting Definitions and Terminology of Nutritional Disorders in Older People with Cancer. Nutrients. 2021;13:
10. Ferrannini E, Simonson DC, Katz LD, Reichard G, Jr., Bevilacqua S, Barrett EJ, et al. The disposal of an oral glucose load in patients with non-insulin-dependent diabetes. Metabolism. 1988;37:79-85.
11. Mueckler M, Caruso C, Baldwin SA, Panico M, Blench I, Morris HR, et al. Sequence and structure of a human glucose transporter. Science. 1985;229:941-5.
12. Birnbaum MJ, Haspel HC, Rosen OM. Cloning and characterization of a cDNA encoding the rat brain glucose-transporter protein. Proceedings of the National Academy of Sciences of the United States of America. 1986;83:5784-8.
13. Kaestner KH, Christy RJ, McLenithan JC, Braiterman LT, Cornelius P, Pekala PH, et al. Sequence, tissue distribution, and differential expression of mRNA for a putative insulin-responsive glucose transporter in mouse 3T3-L1 adipocytes. Proceedings of the National Academy of Sciences of the United States of America. 1989;86:3150-4.
14. James DE, Strube M, Mueckler M. Molecular cloning and characterization of an insulin-regulatable glucose transporter. Nature. 1989;338:83-7.
15. Fukumoto H, Kayano T, Buse JB, Edwards Y, Pilch PF, Bell GI, et al. Cloning and characterization of the major insulin-responsive glucose transporter expressed in human skeletal muscle and other insulin-responsive tissues. The Journal of biological chemistry. 1989;264:7776-9.
16. Charron MJ, Brosius FC, 3rd, Alper SL, Lodish HF. A glucose transport protein expressed predominately in insulin-responsive tissues. Proceedings of the National Academy of Sciences of the United States of America. 1989;86:2535-9.
17. Birnbaum MJ. Identification of a novel gene encoding an insulin-responsive glucose transporter protein. Cell. 1989;57:305-15.
18. Lin JW, Huang YM, Chen YQ, Chuang TY, Lan TY, Liu YW, et al. Dexamethasone accelerates muscle regeneration by modulating kinesin-1-mediated focal adhesion signals. Cell Death Discov. 2021;7:35.
