# Supplementary figures and images for: Sarcopenia‐related changes in serum GLP‐1 level affect myogenic differentiation
Source: J Cachexia Sarcopenia Muscle. 2024 Jun 26;15(5):1708–21. doi: 10.1002/jcsm.13524 (PMC11446708; doi:10.1002/jcsm.13524)

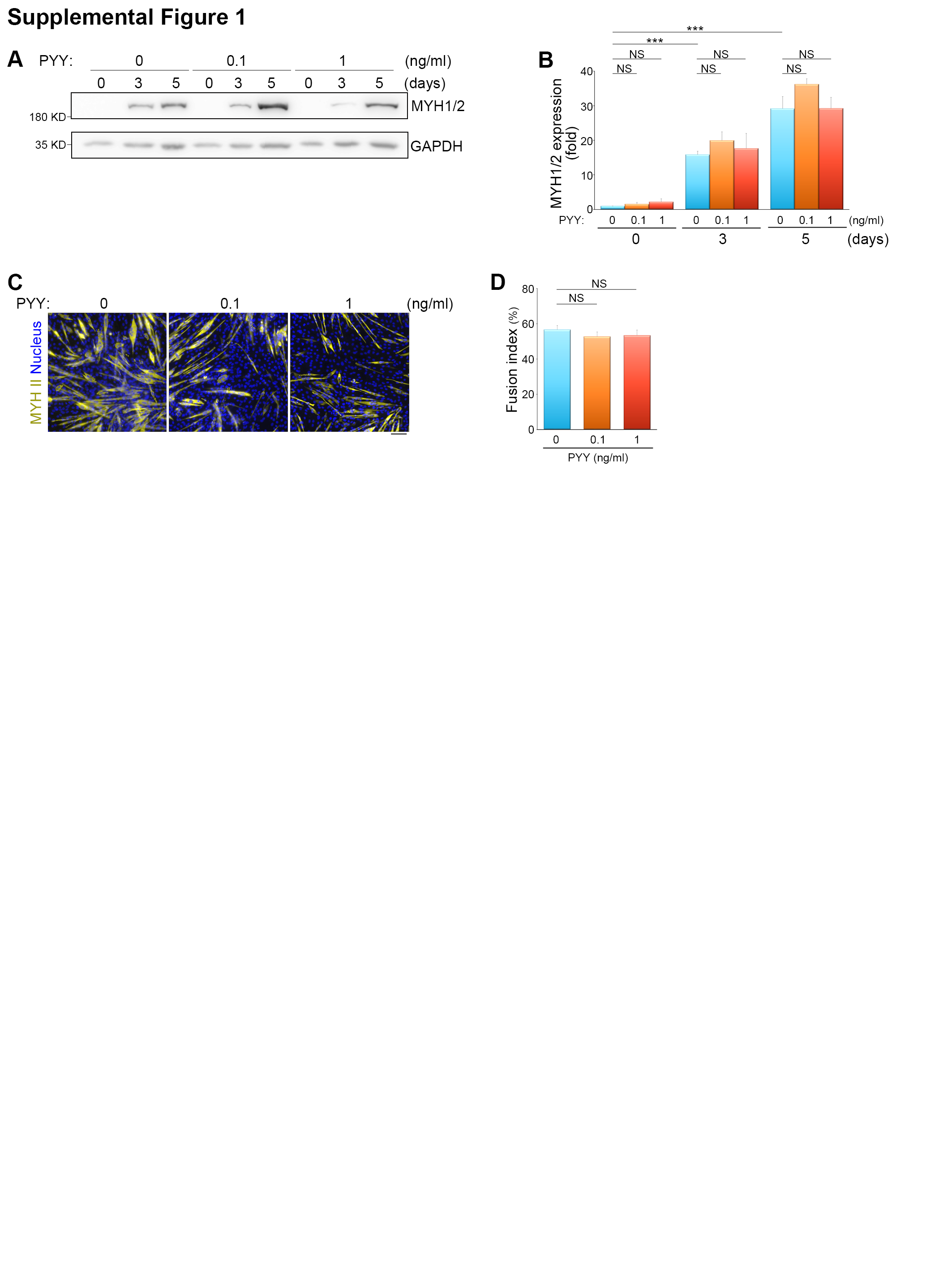

Supplement: Supplementary file 2 — Figure S1. Consistent PYY treatment does not affect the myogenic program in myoblasts. (A) Effects of consistent PYY treatment on myogenic differentiation. C2C12 cells were treated with a myogenic induction medium containing the indicated concentration of PYY for 0, 3 and 5 days and analysed by Western blotting using antibodies against MYH1/2 and GAPDH. (B) The ratio of MYH1/2 to GAPDH is shown as fold. Data are mean ± s.e.m (n = 3 independent experiments). ***P < 0.001; NS, no significance. (C) Effects of consistent PYY treatment on myocyte fusion. C2C12 cells were treated with a myogenic induction medium containing the indicated concentrations of PYY for 5 days and immunostained for myosin 4 (yellow; to visualize 10 MYH II + myotubes) and DAPI (blue; to visualize nucleus). Scale bar, 100 μm. (D) Fusion index, calculated as the percentage of nuclei (≥ 3) in MYH II + cells, as shown in (C). Data are mean ± s.e.m (0 ng/ml PYY: n = 13 independent fields [total 537 MYH II + cells counted]; 0.1 ng/ml 13 n = 10 independent fields [total 368 MYH II + cells counted]; 1 ng/ml PYY: n = 6 independent fields [total 339 MYH II + cells counted], from 3 independent experiments). NS, no significance. [file JCSM-15-1708-s002.tif]

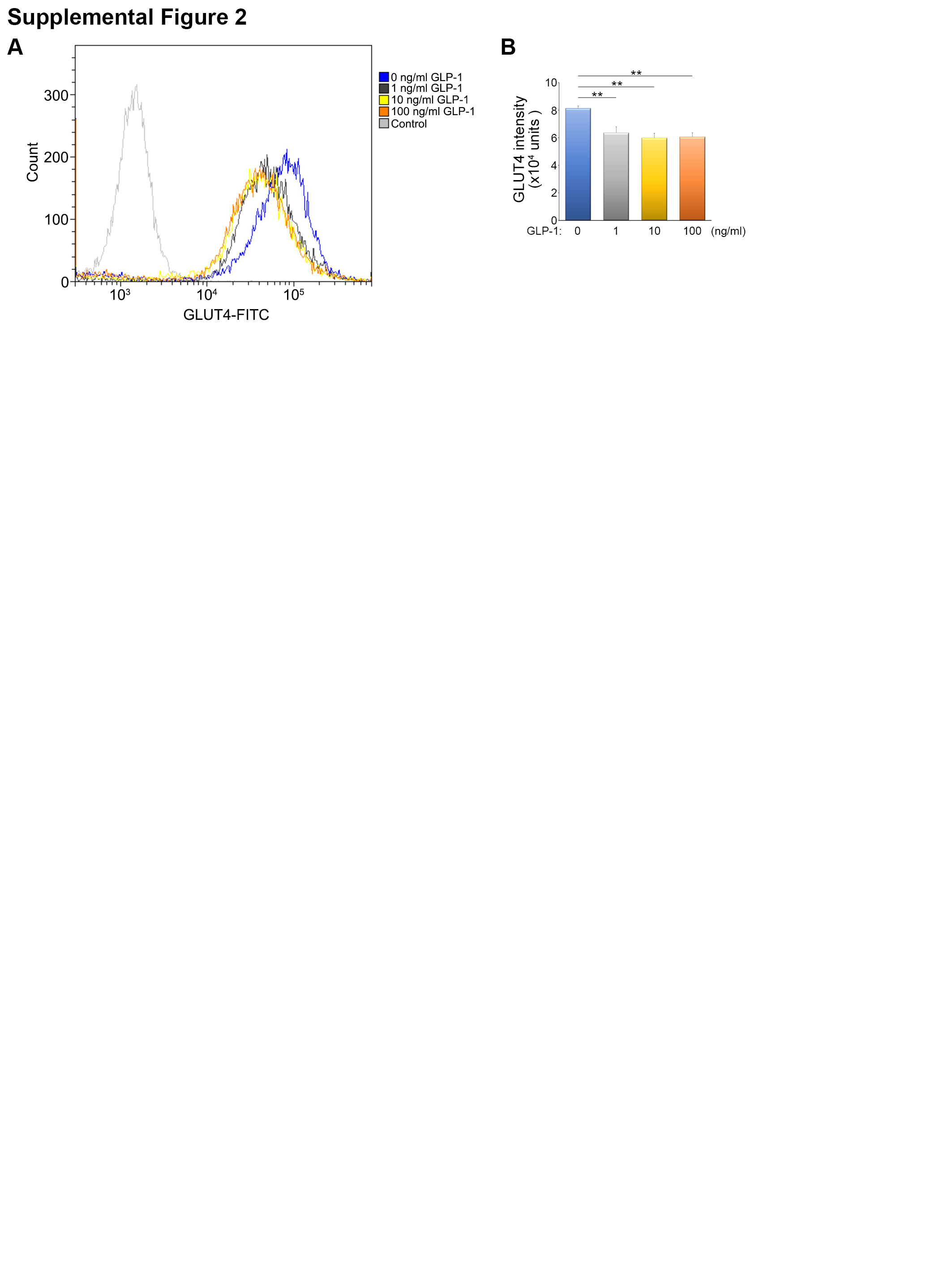

Supplement: Supplementary file 3 — Figure S2. Consistent GLP‐1 treatment inhibits GLUT4 membrane translocation during myogenic differentiation. (A and B) Effects of consistent GLP‐1 treatment on membrane expression of GLUT4. Cells were treated with a myogenic induction medium containing the indicated concentration of GLP‐1 for 5 days. Subsequently, flow cytometry analysis was conducted using antibodies against GLUT4 or buffer alone (Control), followed by labelling with the Alexa Fluor 488‐conjugated secondary antibody. (A) The representative flow cytometry plots. (B) The average GLUT4 intensity. Data are mean ± s.e.m (n = 4 independent experiments). **P < 0.01. [file JCSM-15-1708-s001.tif]
